# Additional Value of FDG-PET/MRI Complementary to Sentinel Lymphonodectomy for Minimal Invasive Lymph Node Staging in Patients with Endometrial Cancer: A Prospective Study

**DOI:** 10.3390/diagnostics14040376

**Published:** 2024-02-09

**Authors:** Matthias Weissinger, Lidia Bala, Sara Yvonne Brucker, Stefan Kommoss, Sascha Hoffmann, Ferdinand Seith, Konstantin Nikolaou, Christian la Fougère, Christina Barbara Walter, Helmut Dittmann

**Affiliations:** 1 Department of Diagnostic and Interventional Radiology, University Hospital Tuebingen, 72076 Tuebingen, Germany; 2 Department of Nuclear Medicine and Clinical Molecular Imaging, University Hospital Tuebingen, 72076 Tuebingen, Germanychristian.lafougere@med.uni-tuebingen.de (C.l.F.); helmut.dittmann@med.uni-tuebingen.de (H.D.); 3 Department of Women’s Health, University Hospital Tuebingen, 72076 Tuebingen, Germany; sara.brucker@med.uni-tuebingen.de (S.Y.B.);; 4 Gynecologic Oncology, Diakonie-Hospital Schwäbisch Hall, 74523 Schwäbisch Hall, Germany; 5 Image-Guided and Functionally Instructed Tumor Therapies (iFIT)-Cluster of Excellence, Eberhard Karls University, 72076 Tuebingen, Germany; 6 German Cancer Consortium (DKTK), Partner Site Tuebingen, 72076 Tuebingen, Germany

**Keywords:** endometrial cancer staging, FDG PET/MRI, ^99m^Tc-Nanocolloid SPECT/CT, micrometastases, N-staging, para-aortic lymph node metastases, sentinel lymph node

## Abstract

Background: Lymph node metastases (LNM) are rare in early-stage endometrial cancer, but a diagnostic systematic lymphadenectomy (LNE) is often performed to achieve reliable N-staging. Therefore, this prospective study aimed to evaluate the benefit of [18F]-Fluorodeoxyglucose (FDG) PET/MRI complementary to SPECT/CT guided sentinel lymphonodectomy (SLNE) for a less invasive N-staging Methods: 79 patients underwent a whole-body FDG-PET/MRI, SLN mapping with ^99m^Tc-Nanocolloid SPECT/CT and indocyanine green (ICG) fluoroscopy followed by LNE which served as ground truth. Results: FDG-PET/MRI was highly specific in N-staging (97.2%) but revealed limited sensitivity (66.7%) due to missed micrometastases. In contrast, bilateral SLN mapping failed more often in patients with macrometastases. The combination of SLN mapping and FDG-PET/MRI increased the sensitivity from 66.7% to 77.8%. Additional SLN labeling with dye (ICG) revealed a complete SLN mapping in 80% (8/10) of patients with failed or incomplete SLN detection in SPECT/CT, reducing the need for diagnostic systematic LNE up to 87%. FDG-PET/MRI detected para-aortic LNM in three out of four cases and a liver metastasis. Conclusions: The combination of FDG-PET/MRI and SLNE can reduce the need for diagnostic systematic LNE by up to 87%. PET/MRI complements the SLN technique particularly in the detection of para-aortic LNM and occasional distant metastases.

## 1. Introduction

Uterine cancer is the fourth most prevalent female cancer within the European Union (EU) witnessing over 70,000 new cases in 2020 across the 27 EU member states; on the global scale, it is the seventh-most common female cancer worldwide [1,2,3]. Besides initial tumor infiltration and tumor genetics, the presence of lymph node metastases (LNM) and distant metastases plays a pivotal role in determining treatment strategies and has significant impact on overall survival [4,5].

Unlike many other solid tumors, the staging of endometrial carcinoma follows the Fédération Internationale de Gynécologie et d’Obstétrique (FIGO) classification prioritizing clinical examination and molecular classification over imaging modalities [5,6,7]. Since patients with a higher risk for lymph node metastases could benefit from an intensified surgical intervention, while patients with a more favorable molecular subtyping may require less aggressive therapy approaches, the integration of preoperative imaging and molecular classification plays an important role [4,5,7]. Because lymph node (LN) staging occurs during surgery, the extent and stage of the disease are often unclear beforehand, making it difficult to determine the optimal therapy and further treatment [4,5,7]. Furthermore, uterine lymphatic drainage is highly variable in endometrial cancer. It can metastasize to either side of the pelvis and even skip the iliac LN region and metastasize directly to the retroperitoneal space via the pelvic-infundibular ligament pathway [5,8]. Consequently, extensive diagnostic iliac and para-aortic lymphonodectomy (LNE) is often performed for precise N-staging [4,5]. During LNE, approximately 50 LN are resected, which increases the risk of intraoperative and postoperative complications such as chronic lymphedema [4,9]. In 1996, sentinel lymph node excision (SLNE) was introduced as a less invasive yet comparably accurate method for N-staging of endometrial carcinoma [10]. SLNE is considered for N-staging in low-to-intermediate-risk endometrial cancer as an alternative to systematic LNE according to the current ESMO guidelines [5,11,12].

For SLN mapping, substances like blue dye, indocyanine green (ICG) or radiolabeled particles such as ^99m^Tc (Tc)-Nanocolloid are injected into the cervix to trace the individual lymphatic drainage pathways [4,5]. If the first LN of the lymphatic drainage is considered tumor-free during rapid section, further LN resection can usually be omitted [13]. Unfortunately, bilateral SLN detection fails in about 10–20% due to high venous outflow, improper injection, or destruction of the lymphatic drainage pathways by tumor, as recent reports have shown [14,15,16,17]. Moreover, the SLN technique has its limitations in detecting para-aortic LNM, especially in the absence of pelvic LNM, which would indicate para-aortal LNE [4,6,17]. Those so called “isolated para-aortic LNM” occur in approximately 0.5 to 3.8% of patients [5,8]. As SLN marking is performed during surgery, there is no possibility to repeat the examination or resort to imaging. In case of incomplete or failed SLN detection, the escalation to a systematic pelvic LNE is recommended except for pT1a G1-2 tumors [4].

An alternative to the surgical staging might be magnetic resonance imaging (MRI). MRI is recommended for locoregional LN staging, particularly in advanced disease and prior to primary radiotherapy, but it comes with limited sensitivity of 43.5–72% [4,5,18,19,20]. Combining MRI with [18F]-fluorodeoxyglucose-positron emission tomography (FDG-PET) offers potential, yet FDG-PET/MRI data are limited, and its use is mostly reserved for clinical trials, high-risk patients, and the evaluation of tumor recurrence [4,5,18,21,22].

Therefore, this study aimed to evaluate the additional value of FDG-PET/MRI imaging to SLNE for minimally invasive N-staging in a prospective trial.

## 2. Materials and Methods

### 2.1. Cohort

**Figure 1:**
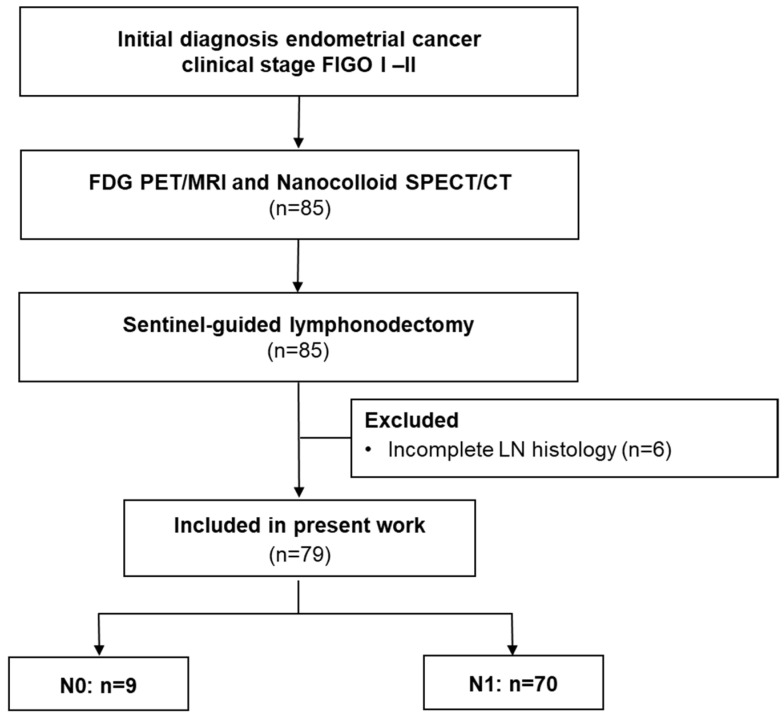
CONSORT flow diagram. 85 patients met the inclusion criteria. Six dropouts occurred due to incomplete histology.

From February 2017 to April 2022, 79 patients with an initial diagnosis of endometrial cancer and clinically suspected FIGO I–II stage were enrolled in this prospective study as presented in detail in the Consolidated Standards of Reporting Trials (CONSORT) flow diagram (Figure 1). This prospective study was approved by the institutional review board (registry No.173/2015BO01) and is listed in the German Clinical Trial Register (DRKS: DRKS00014346) [23]. All patients signed informed consent.

All patients underwent a whole-body FDG-PET/MRI and a preoperative SPECT/CT-guided SLN mapping, followed by an intraoperative SLN detection using a gamma probe and a minimally invasive lymphonodectomy. The average patient age at the time of the PET/MRI was 61 years (range: 25–83 years), with a BMI of 29.0 (range: 19.1–43.4), average weight of 78 kg (range: 49–129 kg), and a height of 164 cm (range: 150–177 cm). The time interval between the FDG-PET/MRI and LN histology was 16.1 ± 11.1 days.

### 2.2. PET/MRI Protocol

All examinations were performed with one PET/MRI device (Biograph mMR^®^, Siemens Healthineers, Erlangen, Germany). The tracer used was [18F]Fluorodeoxyglucose (FDG), which was produced in compliance with governmental radiopharmaceutical safety regulations at our institute’s radiopharmaceutical facility.

All patients were asked to fast for a minimum of six hours before receiving FDG (242.9 ± 18.8 MBq) injections contingent on blood sugar levels below 140 mg/dL. Additionally, all patients received 20 mg butylscopalaminin intravenously, except where contraindicated, to minimize intestinal motility.

Simultaneous FDG-PET and MRI acquisitions covered the region from mid-thigh to the skull base, with a minimum acquisition time of 4 min per bed position. After the whole-body scan, gadolinium-enhanced T1w and pelvic T2w sequences were acquired with a weight-adjusted PET acquisition time of approximately 15 min. Details of the MRT sequences are given in Table 1. The evaluation of the FDG-PET/MRI images was performed by board-certified nuclear medicine and radiology experts, each with a minimum of five years of experience in FDG-PET and MRI imaging.

### 2.3. Sentinel Lymph Node Marking and Mapping

For the SLN mapping, approximately 200 MBq ^99m^Tc-(Tc) Nanocolloid (Nanocoll^®^, GE Healthcare, Braunschweig, Germany) diluted with 0.9% NaCl to a volume of 0.8 mL was injected intracervical at three, six, nine and twelve o’clock positions. SLN imaging was performed with one hybrid SPECT/CT device equipped with two camera heads in H-mode (Discovery 670 Pro^®^, GE Healthcare, Chicago, IL, USA) three to five hours after the Tc-Nanocolloid injection. For attenuation correction and anatomical localization of the SPECT data, a low-dose CT scan was initially performed with an integrated 16-slice CT scanner employing a dose-modulation system (120 kV, 10–80 mAs, OptiDose^®^, GE Healthcare, Chicago, IL, USA). The gantry rotated at 0.8 s, with a table feed of 20 mm per gantry rotation. A monomeric, nonionic contrast agent (90 mL Ultravist 370^®^, Bayer Vital GmbH, Leverkusen, Germany) was administered, except in cases of contraindications.

The SPECT acquisition followed immediately after the low-dose CT. The field of view covered inguinal to para-aortic (up the renal artery branch) LN regions. SPECT acquisition parameters included an energy window of 140.5 keV ± 10%, a 128 × 128 matrix, 30 angular steps at 6° intervals, a 15 s acquisition time per step, and a pixel size of 4.42 × 4.42 mm. SPECT data were reconstructed using an OSEM iterative protocol (2 iterations, 10 subsets) as previously described [14].

SLN SPECT/CT images were jointly reviewed by two nuclear medicine experts, each with over a decade of expertise in pelvic SLN imaging. A SLN was characterized as the presence of focal tracer uptake on SPECT within a plausible anatomical region. A successful SLN mapping was defined as the identification of at least one clearly discernible SLN per hemipelvis; complete SLN mapping was defined as successful SLN mapping in both hemipelves.

### 2.4. Sentinel Lymphonodectomy

Surgical staging was performed laparoscopically the morning after the Tc-nanocolloid injection. At start of the surgery, dye (indocyanine green = ICG, Verdye^®^ 5 mg, Diagnostic Green GmbH, Kirchheim, Germany) was injected intracervically at the three and nine o’clock positions (1 mL per injection).

Intraoperatively, SLNs were localized with laparoscopic gamma probe (Neoprobe^®^, Models 1017 and 1100, Devicor Medical Products Inc., Cincinenati, OH, USA) and by direct visualization (ICG Optics, Storz; ICG Rubina System^®^, Storz; Ligh source: D-light^®^, Storz, Tuttlingen, Germany). The SLNs were individually resected, carefully labeled, and immediately sent for rapid tissue section analysis.

All patients underwent a bilateral LNE tailored to their tumor stage, which included the removal of SLNs and at least macroscopically suspicious LNs. In cases of intraoperatively identified advanced tumor stage or upon histopathological confirmation of LNM through frozen section analysis, additional para-aortic LNE was performed. The SLNs underwent comprehensive histological processing, including ultrastaging with 200 µm slices.

### 2.5. Statistical Analysis

Statistical analysis was performed with SPSS^®^ Statistics 28.0 software (IBM Inc., Armonk, NY, USA) and MedCalc 18.10 (MedCalc Software Ltd., Ostend, Belgium). Test performance metrics were computed using contingency tables. A *p*-value < 0.05 was considered statistically significant.

## 3. Results

### 3.1. Tumor Histology and Prevalence of LNM

In total, lymphonodectomy was performed in 79 patients or 158 hemipelves.

Tumor stages pT1 and pT2 were predominant in the cohort, although imaging and histology upstaged four patients to pT3 tumors (see Table 2). Tumor grade was predominantly G1 followed by G2 (G1: *n* = 47, G2: *n* = 16, G3: *n* = 10, Gx: *n* = 6).

The prevalence of LNM was 11.3% per patient (9/79) and 7.0% per hemipelvis (11/158). Out of 9 patients, 4 presented exclusively nodal micrometastases smaller than 2 mm. LNM was observed in every tumor stage—ranging from pT1a to pT3b—and across all tumor grades, as detailed in Table 2. Para-aortic LNM were identified in 4 out of the 24 patients who underwent para-aortic LNE, with one of them presenting isolated para-aortic LNM.

### 3.2. FDG-PET/MRI

The prospective FDG-PET/MRI reading by expert consensus revealed a high accuracy on patient level (75/79; 94.9%, 95% CI: 87.5–98.6%) as well as on hemipelvis level (152/158; 96.2%, 95% CI: 91.9–98.6%). In detail, FDG-PET/MRI demonstrated high specificity in detecting LNM with a specificity rate of 98.6% (69/70; 95% CI: 92.3–99.9%) but exhibited a moderate sensitivity of 66.7% (6/9; 95% CI: 9.9–65.1%) at the patient level. At the hemipelvis level, results were comparable with a specificity of 98.0% (144/147; 95% CI: 94.2–99.6%) and a sensitivity of 72.7% (8/11, 95% CI: 39.0–94.0%). The size of LNM was a critical factor for detectability in FDG-PET/MRI, with sensitivity dropping to 25.0% for micrometastases (less than 2 mm) compared to 83.3% for LNM of 2 mm diameter or larger.

The PPV and NPV of FDG-PET/MRI for LNM detection were 85.7% (6/7, 95% CI: 44.8–97.8%) and 95.8% (69/72, 95% CI: 90.1–98.3%), respectively, at the patient level and 72.7% (8/11, 95% CI: 45.1–89.6%) and 98.0% (144/147, 95% CI: 94.8–99.2%), respectively, at the hemipelvis level.

Para-aortic LNM were detected with PET/MRI in 3 out of 4 patients in the subgroup of 24 patients with additional para-aortic lymphonodectomy, resulting in a sensitivity of 75.0% (CI: 19.4–99.4) and a specificity of 100% (CI: 95.2–100.0%). FDG-PET/MRI revealed a hepatic metastasis in one patient, which was histologically confirmed via biopsy.

### 3.3. Tc Nanocolloid SLN Marking

In SLN mapping with SPECT/CT, at least one SLN was imaged in 88.6% (70/79) of all patients or in 68.4% (108/158) of all hemipelves. Complete SLN mapping in both hemipelves was successful in 49.4% (39/79) of the patients. In 12.7% of all patients (10/79), SLN mapping failed in both hemipelves. The occurrence of unilateral or failed SLN mapping was higher in patients with suspicious FDG-positive lymph nodes, with a rate of 71.4% (5/7) in those with positive FDG-PET/MRI findings compared to 36.1% (26/72) in those with negative FDG-PET/MRI results. The presence of LNM was higher in patients with single-sided or failed SLN mapping in SPECT/CT (15.0%, 6/40) than in patients with complete mapping on both hemipelves (7.7%, 3/39). Successful para-aortic SLN mapping in SPECT/CT was achieved in 12 out of 79 patients; however, none of them showed tumor cells upon ultra-staging. Additionally, no para-aortic LNM were detected in SPECT/CT imaging.

### 3.4. ICG SLN Marking

Sixty-one patients underwent additional perioperative SLN marking with ICG as part of best clinical practices at our center and were retrospectively included in the analysis. Among these patients, intraoperative laparoscopic SLN detection was complete in both hemipelvis in 77.0% (47/61) of the cases, incomplete and unilateral in 16.4% (10/61), and failed in 6.6% (4/61). None of the para-aortic LNM of the four patients with retroperitoneal nodal involvement were marked with ICG.

### 3.5. Combined FDG-PET/MRI and SLN Protocol

**Figure 2:**
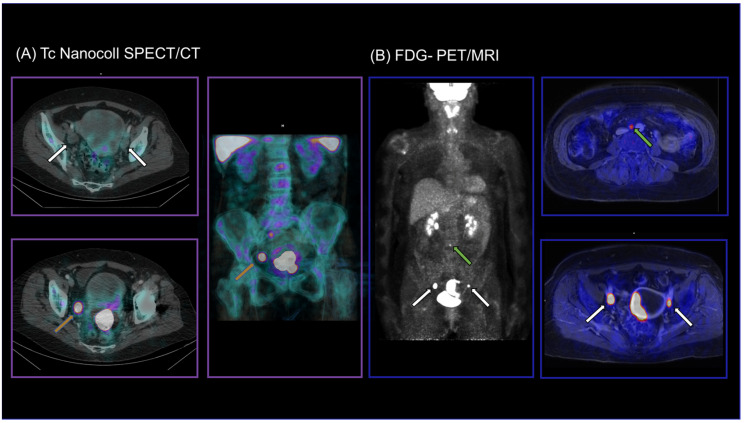
SLN-SPET/CT (**A**) and FDG-PET/MRI (**B**) images of a 65-year-old patient with initial diagnosis of endometrial cancer. LN mapping was incomplete in SPECT/CT with unilateral tracer accumulation on the right hemipelvis only (orange arrows). FDG-PET/MRI detected bilateral iliac external LNM (white arrows) as well as para-aortic LNM (green arrow), which were removed and confirmed histologically (pT3a, pN1, G3).

Adding SLN to FDG-PET/MRI (FDG-PET/MRI positive and complete SLN mapping with SPECT/CT and/or ICG) increased the sensitivity from 66.7% (6/9) to 77.8% (7/9) compared to FDG-PET/MRI alone on the patient level. On the hemipelvis level, a combined protocol of FDG-PET/MRI and SLN increased the sensitivity from 72.7% (8/11) to 82.2% (9/11). In detail, all LNM could be detected on the right hemipelvis (5/5), whereas two remained undetected on the left hemipelvis (4/6). A representative case is presented in Figure 2.

**Figure 3:**
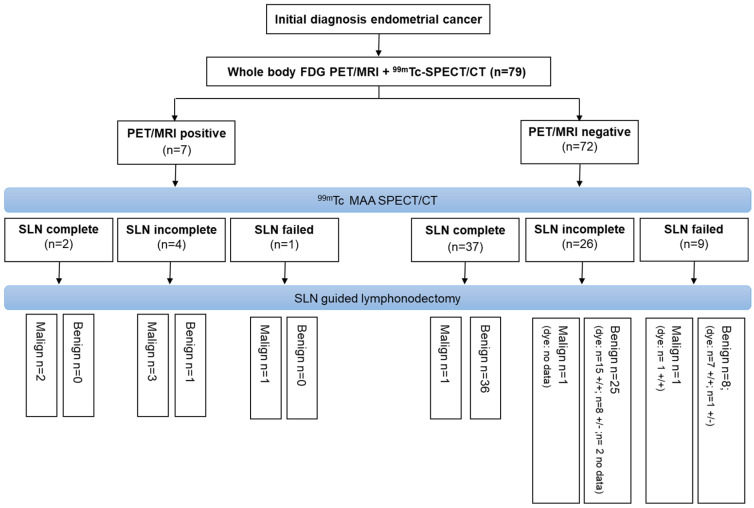
Flow chart illustrating a procedure for minimally invasive N-staging where FDG-PET/MRI is the initial step followed by SLN mapping with SPECT/CT. In case of incomplete SLN mapping, intraoperative ICG marking is performed. Bilateral SLN detection with ICG is denoted as “+/+”, while unilateral SLN detection is indicated as “+/−”.

The additional perioperative SLN labeling with ICG revealed a complete SLN marking with SLN in both hemipelvis in 80% (8/10) of patients with failed SLN mapping in SPECT/CT. An overview of the combined protocol is visualized in Figure 3.

For the detection of para-aortic LNM, FDG-PET/MRI was leading with a sensitivity of 75%, which could not be improved by the SLN technique with either SPECT/CT or ICG staining.

## 4. Discussion

Sentinel lymph node excision is becoming increasingly important for minimally invasive lymph node staging in endometrial cancer. However, it has its limitations in the evaluation of para-aortic SLNs and in case of incomplete SLN mapping. Although FIGO 2023 indicates a notable shift from morphologic to molecular classification, this does not diminish the importance of the imaging and assessment of lymph nodes. Therefore, our objective was to investigate potential additional benefits of a combined protocol using FDG-PET/MRI along with the SLNE. Our data indicate that FDG-PET/MRI is highly specific for the detection of pelvic and para-aortic LNM. However, current PET/MRI devices do not seem to be sensitive enough to detect small micrometastases. In contrast, the SLN technique revealed a high detection rate for small pelvic SLNs but a low sensitivity for para-aortic LNM. Our findings indicate that the proposed combined protocol improves sensitivity for both pelvic and para-aortic LNM and has the potential to reduce the need for diagnostic systematic LNE. Additionally, FDG-PET/MRI enables the detection of extralymphatic metastases.

The reported sensitivity of PET/MRI for LNM detection of 98.6% is in line with previous FDG-PET/MRI studies (90.5–92.9%) and FDG-PET/CT studies (92.7–94.7%) in high-risk patients with prevalence for LNM of up to 20% [24,25,26,27]. However, sensitivity of FDG-PET/MRI drops in case of micrometastases due to its limited spatial resolution of about 5 mm [28]. Overall sensitivity in our study was lower (66.7%) than reported in previous PET/MRI trials [24] and overall in line with PET/CT study results (63.0–68.7%) [26,27]. A reason might be the ultrastaging, which was defined as the gold standard in our study. Ultrastaging, which includes the processing of the whole lymph node in 200 µm layers is known to uncover more micrometastases, which results in an upstage of about 5% of the patients. The higher prevalence of micrometastases in our cohort compared to previous PET/MRI studies thus may have significant impact on sensitivity [17,24]. Considering only macrometastases ≥2 mm, the sensitivity increases to 83.3% which is comparable to data in literature, involving small number of patients (85.7%, *n* = 7 to 100.0%, *n* = 2) [24,25]. The clinical relevance of micrometastases < 2 mm found in ultrastaging remains unclear. In cases of isolated tumor cells, the relevance might even be irrelevant [11,29]. However, most experts and current guidelines classify micrometastases as lymph node involvement, prompting recommendations for additional adjuvant therapy [4,6,30].

The combined protocol, as proposed here, which includes SPECT/CT-based SLN mapping one day prior to surgery, was easily integrated into the clinical workflow. However, the rate of complete SLN mapping in both hemipelves with SPECT/CT was slightly lower than reported in the literature [31]. It is noteworthy that there was a higher prevalence of LNM in cases of incomplete unilateral or failed SLN mapping compared to cases with complete SLN mapping (15.0% vs. 7.7%). We also observed a similar effect in our previous study on cervical cancer when using deep intracervical Tc-Nanocolloid injection, suggesting that this phenomenon may not solely be attributed to high venous drainage or poor injection technique [14,15]. As previously reported, LNM here could negatively affect lymphatic drainage and consequently nanocolloid and ICG efflux from injection depots, which may be a factor in the lower detection rate reported in this study [14]. Complete bilateral SLN mapping is of high clinical relevance especially in intermediate risk carcinomas as incomplete SLN mapping may result in an escalation to systematic LNE on the respective hemipelvis, according to current guidelines [4,6].

Additional perioperative ICG injection in patients with incomplete SLN mapping in SPECT/CT might be a way to improve the SLN detection rate. The additional value of ICG has also been described in the SLN metanalysis by Cormier et al. [17]. Adding ICG to Tc-Nanocolloid turned 80% of our patients with failed or incomplete SPECT/CT SLN mapping to complete SLN mapping. An explanation might be the lower molecular weight of ICG, which may be less affected by tumor-induced changes in uterine lymphatic drainage [14,32]; however, the specific pharmacokinetic must be further investigated.

Furthermore, it has to be considered that ICG retains its fluorescent signal in lymph nodes for an extended period, which can result in the detection of multiple fluorescent nodes [32]. This extended duration may make it challenging to distinguish true SLNs from echelon lymph nodes [32]. In cases with a significant time gap between injection and exploration, such as prolonged surgical procedures such as adhesiolysis, multiple lymph nodes may become fluorescent, potentially leading to a broader LNE instead of a specific SLNE [32].

In the evaluation of para-aortic LNM, PET/MRI revealed a sensitivity of 75%, which is consistent with the reported literature ranges for PET/CT [22]. Consequently, the combination with PET/MRI adds a high benefit to the SLN technique was shown to achieve only limited detection rates of about 40%. The assessment of para-aortic LNs holds particular significance in endometrial carcinoma as isolated para-aortic LNMs may be undetected during operative procedure in cases with negative pelvic lymphodonectomies. The escalation to para-aortic LNE is subject to the judgment and experience of the surgeon according to the NCCN guidelines [8,33].

Due to the high specificity of PET, consideration may be given to de-escalating from systematic lymphadenectomy to biopsy in cases of PET-positive LNs (especially para-aortic LNs). For patients with intermediate risk and non-suspicious LN status in PET, current data suggest that SLNE may be sufficient for N-staging. SPECT/CT based SLN mapping provides preoperative SLN status assessment, making it easier to consider supplementary SLN marking with ICG in cases of incomplete mapping. In our cohort, the proposed workflow would have reduced the number of patients undergoing diagnostic systematic LNE from an initial 79 patients to 72 patients with negative PET further down to 35 patients with incomplete or failed Tc-Nanocolloid SPECT/CT and ultimately to 10 patients (13%) with weak or imperfect ICG staining.

Furthermore, the hereby-presented imaging-based staging approach could be particularly useful for patients with low-risk endometrial cancer desiring offspring. As surgical treatment is aimed to be avoided to preserve fertility in these patients, sufficient imaging is required in addition to morphological assessment and molecular risk classification [34,35].

## 5. Limitations

Although this trial included the largest cohort of endometrial cancer patients N-staged with FDG PET/MRI to date, the number of LNMs remains low due to the limited prevalence of LNMs in early-stage endometrial cancer, which in turn results in limited statistical power. Moreover, the very low numbers of para-aortic LNMs (*n* = 4) and distant metastases (*n* = 1) limited the evaluation of the diagnostic performance of FDG-PET/MR for staging of para-aortic or distant metastases.

Furthermore, it should be noted that this study is a prospective but unicentric trial. It is important to verify the generalizability of these results to other oncological centers. A sub-evaluation of the study included clinically indicated ICG SLN labeling. This additional ICG labeling was performed outside of this study protocol. Nevertheless, the results were compelling enough to warrant inclusion in this analysis. Data in the literature indicate that labeling with ICG generally yields a higher sensitivity than labeling with Tc-Nanocolloid [12]. Whether this is associated with lower specificity and a higher rate of false positive lymph nodes should be investigated further, as this could potentially increase morbidity.

## 6. Conclusions

The combination of PET/MRI and SLNE can reduce the rate of diagnostic systematic LNE by up to 87%. PET/MRI complements the SLN technique, especially in detecting para-aortic LNM and distant metastases, which would change the treatment strategy from surgery to radiochemotherapy. However, given the limited sensitivity of PET/MRI in detecting micrometastases, a combination with SLNE is warranted.

## Figures and Tables

**Table 1:** MRI parameters.

	Slice Thickness (mm)	Acquisition Matrix	In-Plane Resolution (mm^2^)	Repetition Time (ms)	Echo Time (ms)	Flip Angle	Fat Saturation
**Whole body**							
T2w HASTE cor	5	320 × 320	1.5625\1.5625	1200	91	160	
T2w HASTE tra	5	256 × 172	0.785\0.78125	1200	95	160	
T1w GRE (VIBE) tra	3	384 × 234	1.3021\1.3021	3.95	1.23	10	Dixon fat saturation
DWI (b50/800)	6	128 × 104	1.7578\1.7578	2500	52	90	Water excitation
Post KM: T1w GRE (VIBE)	3	320 × 195	1.2813\1.2813	3.93	1.24/2.48	9	Dixon fat saturation
**Pelvis**							
T2w TSE tra.	3	320 × 320	0.78125\0.78125	5760	101	160	
T2w TSE cor.	3	320 × 310	0.78125\0.78125	5880	101	160	
T2w TSE sag.	3	320 × 310	0.625\0.625	5760	101	160	

**HASTE**: **H**alf-Fourier-**a**cquisition single-**s**hot **t**urbo spin-**e**cho. **VIBE**: **v**olume-**i**nterpolated **b**reath-hold **e**xamination.

**Table 2:** Tumor stages and prevalence of lymph node metastases.

Tumor Stage		Patients	Grading (Number of Pelvic LNM in Brackets)			
			G1	G2	G3	Gx
**pT1**	pT1a	51	40 (0)	7 (0)	4 (1)	-
	pT1b	17	5 (3)	7 (0)	2 (1)	3 (0)
**pT2**	pT2a	6	2 (0)	1 (1)	2 (0)	1 (0)
**pT3**	pT3a	3	-	1 (0)	1 (1)	1 (1)
	pT3b	1	-	-	1 (1)	-
**pTx**		1	-	-	-	1 (0)

Overview of tumor grade distribution and the presence of lymph node metastases according to tumor stage. In one patient, tumor stage and grading could not be conclusively assessed histopathologically based on the obtained material (pTx).

## Data Availability

Data is contained within the article.
